# Cryoneurolysis: A Comprehensive Review of Applications in Pain Management

**DOI:** 10.7759/cureus.79448

**Published:** 2025-02-22

**Authors:** Saniyah Shaikh, Aqsa R Khan, Sanjoli Saini, Anum Naimat, Chaithanya Amudha, Dhyaan Bannur, Eniola Ajayi, Afrah Rehman, Sukesh Shah, Najiyah M Fakhruddin, Nafrin Kormath, Maneeth Mylavarapu

**Affiliations:** 1 Internal Medicine, Alfaisal University College of Medicine, Riyadh, SAU; 2 Internal Medicine, Foundation University Medical College, Islamabad, PAK; 3 Internal Medicine, Max Healthcare and Indian Institute of Management, Kashipur, IND; 4 Internal Medicine, Fatima Memorial Hospital (FMH) College of Medicine and Dentistry, Lahore, PAK; 5 Medicine and Surgery, Saveetha Medical College and Hospital, Chennai, IND; 6 Basic Sciences, University of Texas at Dallas, Richardson, USA; 7 Research, Henry Ford Hospitals, Detroit, USA; 8 Dentistry, University of Georgia, Tbilisi, GEO; 9 Medicine, American University of Integrative Sciences School of Medicine, Bridgetown, BRB; 10 Internal Medicine, Norman Bethune Medical College, Jilin, CHN; 11 Internal Medicine, Muslim Educational Society (MES) Medical College Hospital, Perinthalmanna, IND; 12 Public Health, Adelphi University, Garden City, USA

**Keywords:** acute pain, cryoablation, cryoneurolysis, oncological pain, wallerian degeneration

## Abstract

Pain management is an integral part of healthcare, and cryoneurolysis, a technique that uses extreme cold to disrupt nerve conduction, has demonstrated potential in managing both acute and chronic pain. It is an alternative for patients who do not respond to traditional pain management therapies. This review examines the efficacy, application, mechanism, limitations, challenges, and advancements in cryoneurolysis. Cryoneurolysis has broad applications, including acute pain management, reducing opioid dependency, and shortening hospital stays. It effectively treats chronic pain conditions like lumbar facet syndrome, phantom limb pain, occipital neuralgia, and, in some cases, unresponsiveness to traditional therapies. Furthermore, it offers values in tumor-induced neuropathies and postoperative pain.

Cryoneurolysis has a favorable safety profile, presenting a low risk of minor complications such as infection and bruising, which resolve with proper care. Comparative studies indicate that cryoneurolysis, which induces Wallerian degeneration, is as effective as cooled radiofrequency ablation (CRFA), which achieves thermal nerve degradation to block pain transmission, particularly in knee osteoarthritis. However, its potential limitations impede widespread adoption, including small sample sizes, study heterogeneity, and the lack of standardized protocols. Future research should focus on large-scale trials, comparative studies with other pain management modalities, and developing standardized guidelines to enhance clinical outcomes.

## Introduction and background

Pain management remains a critical and integral part of health practice and is of great importance to patients themselves, especially those who suffer from chronic pain that has significantly affected the quality of their lives. Traditional methods of pain management include medications, nerve blocks, and surgical interventions, each with variable efficacy and potential side effects. Consequently, the quest continues for newer methods to provide effective, long-lasting relief with fewer risks [1]. One such promising technique is cryoneurolysis, which uses extremely cold temperatures to ease pain by disrupting nerve conduction.

Cryoneurolysis, also known as cryoablation or cryoneuroablation, is the application of cold temperatures to targeted nerves to "freeze" them in an attempt to block pain signals [2]. This technique was introduced to treat acute pain initially but has significantly gained interest as an intervention for those struggling with chronic pain conditions, primarily due to the failure of traditional interventions that resulted in temporary results and further complications. Evidence has shown that cryoneurolysis may offer long-term pain relief without the need for constant pharmaceutical interventions [3]. It is for this reason that it represents a desirable therapeutic opportunity to consider for patients suffering from chronic knee pain, neuropathic pain, and post-surgery-related pain.

Owing to its minimally invasive/non-invasive nature and lasting pain relief over months, cryoneurolysis offers significant advantages over traditional pain management methods [1,4]. Furthermore, cryoneurolysis does not have systemic effects or addiction risks, as seen in long-term opioid use, making it a better pain management option today in the context of the increasing opioid crisis [5].

Cryoneurolysis, with its prolonged analgesic effects, can facilitate early mobilization in postoperative situations, reducing the length of hospitalization. This shows promise in post-operative and acute pain settings. Studies have demonstrated its efficacy in total knee arthroplasties, shoulder surgeries, and thoracotomies [6]. Cryoneurolysis has also been proven to be a valuable alternative to traditional pain management in chronic pain conditions, especially when traditional methods fail. Multiple studies have reported successful pain management for osteoarthritis of the knee and various neuropathic pain syndromes, including trigeminal neuralgia and phantom limb pain [4,7]. Despite its increasing use, cryoneurolysis remains under-researched compared to other modalities. Many questions remain about its long-term effects and optimal application [8]. This review explores the efficacy of cryoneurolysis in pain management, its mechanisms, advantages and limitations, potential challenges, and recent developments in cryoneurolysis.

## Review

Historical overview

Cryoneurolysis first appeared in 460 BC when ancient Egyptians and Greeks, such as Hippocrates, used ice and snow to treat wounds [7]. This pain management technique has evolved with many advancements in recent centuries. For instance, in 1851, an English physician named James Arnott published his method of using ice and salt mixtures to treat nerve pain. In 1899, a Scottish chemical manufacturer named Campbell White used refrigerants for medical purposes [9].

During the 1900s, more modern innovations in cryoneurolysis were introduced, along with the invention of neurosurgery. In 1913, neurosurgeon Dr. Irving S. Cooper designed a liquid nitrogen probe that could reach temperatures of -196°C to treat movement disorders like Parkinson's. Next, Amoils developed an improved version called the cryoprobe, using carbon dioxide and nitrous oxide, which reached -70°C [10]. 

**Figure 1:**
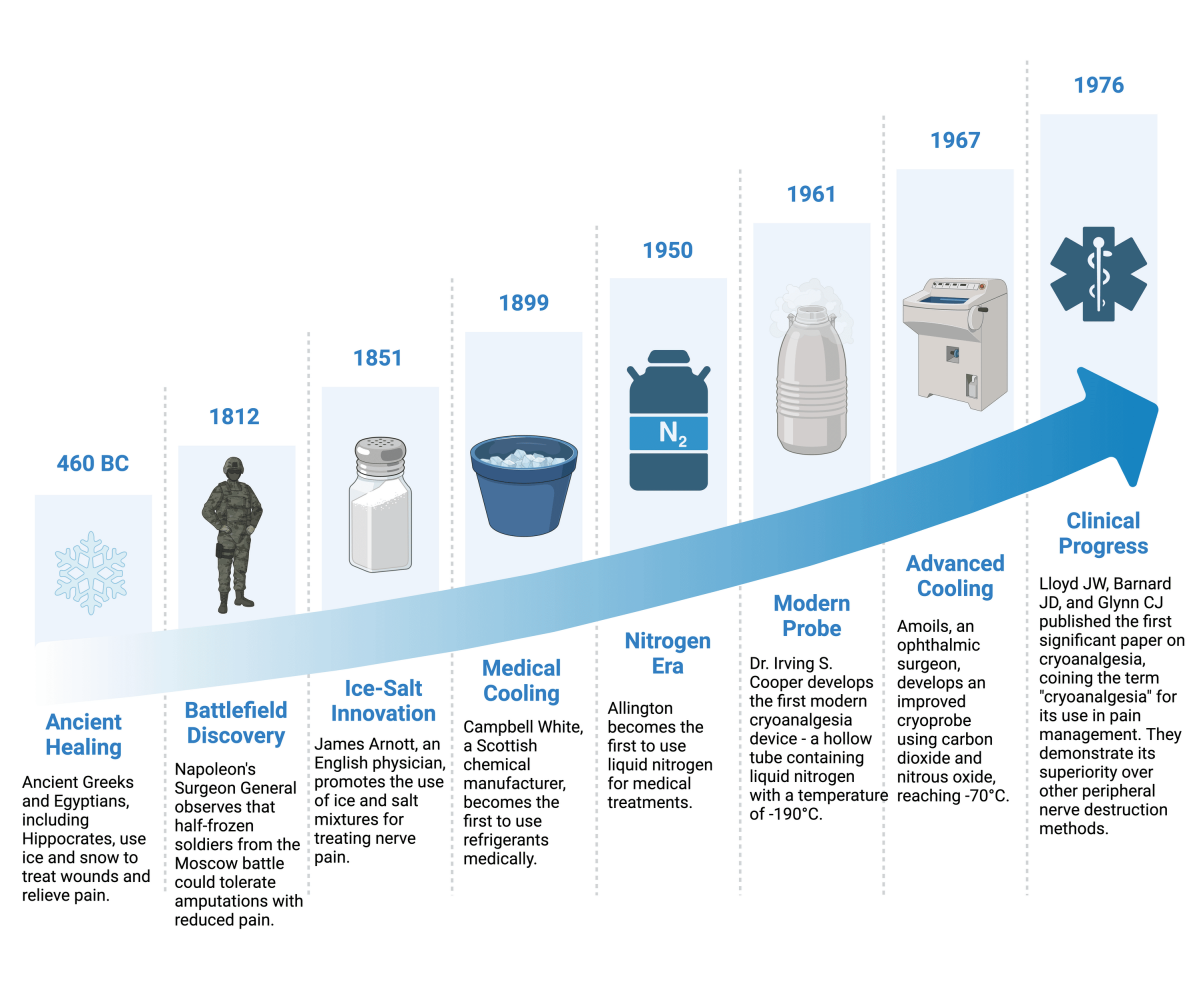
Historical evolution of cryoneurolysis in pain management This timeline depicts how using cold for pain relief has changed over time. Created in BioRender. Mylavarapu, M. (2025) https://BioRender.com/y55z016; Sources: [6,7, 9-12]

These techniques initially required surgical incisions to access target nerves, which limited their use to specific procedures like thoracotomies, tonsillectomies, and herniorrhaphies [11]. However, with the development of percutaneous techniques, the requirement for surgery vanished, and the integration of imaging technologies such as ultrasound, CT, and MRI broadened the scope of cryoneurolysis applications [6]. Overall, this opened up many possibilities in acute and chronic pain management. Figure 1 depicts the historic evolution of cryoneurolysis in pain management [6,7, 9-12].

Cryoablation technology

Cryotechnology advancements have led to handheld cryoprobes, transforming nerve architecture visualization via ultrasound. These devices generate variable ice ball diameters on superficial peripheral nerves. Notable cryoprobes include Wavetip, NeuroTherm, and Baylis Medical, offered in sizes from 1.4 to 2 millimeters. They include integrated nerve stimulators for precise localization and thermistors for tip temperature monitoring. The nerve stimulator is set for sensory (100 Hz) or motor (2 Hz) responses. An introducer is recommended for optimal efficacy, as it isolates current at the probe tip, protects skin during treatments, and facilitates anesthetic infiltration for nerve blockage using fluoroscopy, ultrasonography, and nerve stimulators [1].

The cryoprobe is crucial to cryoneurolysis, utilizing low temperatures (-20°C to -100°C) for three to five minutes to induce axonal degeneration. It has three parts: a cryogen source, a cryo-destructive section, and an insulated handle. The heat of vaporization of gases justifies using them as cryogen sources. The phase diagram of gases indicates achievable temperature values for liquefied gases under standard conditions. For cryoneurolysis, three device types are used to achieve core temperatures from -20°C to -80°C: nitrous oxide, carbon dioxide, and argon-based devices [8].

Devices comprise a cryogen tank, pressure regulator, feed line, pneumatic block, handpiece, and cryo-destructive section or cryoprobe. This is a metallic tube with a central internal channel. The tube is bayonet-shaped in carbon dioxide and argon devices, and the cooling agent circulates through it. Nitrogen devices feature a simple rigid cryoprobe without internal channels; thermal contact with the cooling agent creates a gas pressure drop and adiabatic cooling. An oversized gauge intravenous catheter serves as the introducer, featuring a tapered tip for easy tissue penetration. The stylet can be removed for easier probe insertion. The 2.0 mm probe uses a 12-gauge catheter, while the 1.4 mm probe fits a 14-gauge or 16-gauge catheter [8].

Accurate cryoprobe insertion requires precision to avoid harming nearby neural tissue. Key technical considerations for neural blockade include imaging for correct placement and selective blockade with the iceball. Imaging is advised during contrast medium, or a local anesthetic solution is recommended. Cone-shaped iceballs form 1.5 cm from the cryoprobe tip, with the base away from the probe. The lesion's elongated shape and the probe's proximity to neural structures usually do not risk non-selectivity, as the iceball's basilar surface is oriented away from neural tissue, often in a sigmoid shape along the nerve’s length [13].

Avoid nonselective cold lesions by minimizing freezing times and adjusting the probe’s location if sensory or motor responses occur before pain sensation is lost. Local pressure from the probe on neural tissue should be avoided, confirmed by ensuring free movement of the visible nerve. Continuously monitor nerve stimulation when freezing near sensory and motor nerves. High-frequency ultrasound aids in visualizing the cryoprobe's in vivo position relative to surrounding tissues, especially nerves. With these precautions, a successful block can be achieved at cervical and lumbar nerves, where distinguishing between sensory and motor nerves is anatomically challenging [14].

Extracellular ice formation during freezing disrupts the osmotic balance in pain-sensitive and autonomic nerves innervated by sympathetic fibers, indicating a systematic conceptual expression. Near applied cold temperatures, nerve responses desynchronize, accelerate, and relax [15]. Animal studies of freezing-induced neural ablation and the basis and utility of the cryoneurolysis model are discussed. These studies reveal molecular-level alterations within the ablated nerves and the spontaneous, non-reversible disruption of multiple physiological responses. Cellular manifestations of neural disruption are evident at the cellular, tissue, and extracellular levels [15, 16].

Indications and applications in pain management

Pain is a subjective experience that significantly impacts the quality of life. However, the extent of its impact varies according to the anatomical region or pain site, the system responsible for the perceived pain, and the characteristics, patterns, time, intensity, and etiology of the pain [17]. Hence, personalized treatment approaches would be significantly beneficial. Multiple studies have reported promising results in the utilization of cryoneurolysis for pain management in both acute and chronic pain settings [18-35]. 

Acute pain management 

Cryoneurolysis in peripheral nerve blocks for acute pain has progressed considerably over the years. Initially focused on intercostal nerve ablation for postoperative pain in thoracotomy patients, the indications for cryoneurolysis have broadened to effectively address various types of pain, including, but not limited to, surgical pain, postoperative pain, pain resulting from trauma, neuropathic pain, delivery pain, allodynia, and so forth [8]. These can be applied to a wide variety of clinical settings, such as mastectomy, knee osteoarthritis, total knee arthroplasty, shoulder rotator cuff repair, postherpetic neuralgia, thoracoabdominal aneurysm repair, trauma, allodynia, neuralgias, childbirth, and more, demonstrating its versatility [8]. 

Additionally, its application in non-painful conditions such as obesity, wherein interruption of the vagus nerve has resulted in decreased appetite and/or weight loss, further unlocks its potential for innovative therapeutic applications [36]. Given the advancements in imaging techniques, it is now easier to target specific nerves with precision. The technique's ability to significantly reduce opioid consumption is particularly noteworthy, addressing opioid dependence and the associated side effects. Ultimately, by effectively managing pain with insignificant to absent complications, cryoneurolysis has been shown to improve patient outcomes and contribute to more efficient healthcare delivery, as evidenced by the shortened length of hospital stays and simplified nursing care [8].

Chronic pain management 

Cryoneurolysis of peripheral nerve blocks has also demonstrated effectiveness in managing various chronic pain conditions. For instance, fluoroscopic-guided cryoneurolysis of the obturator nerve has been used to target pain resulting from hip adductor spasticity, providing long-lasting relief. The technique has also shown promise in alleviating phantom limb pain in amputees, chronic peripheral neuropathy pain, and temporomandibular joint pain that does not respond to conventional treatments. In lumbar facet joint syndrome, which accounts for 15% to 54% of lower back pain, CT-guided cryoneurolysis has been shown to decrease depression and pain [26]. 

Additionally, in cases of recalcitrant anterior femoral cutaneous nerve-mediated neuropathic pain, MRI-guided cryoneurolysis has significantly resulted in pain reduction without complications [5,23]. Similarly, pain relief was observed in occipital neuralgia, in which irritation, inflammation, compression, or injury to the occipital nerves causes severe chronic paroxysmal pain [30]. Cryoneurolysis has also proven effective in sports medicine for treating chronic pain conditions like myalgias [37]. Recently, it has also been indicated in treating persistent orofacial pain following dental procedures by targeting alveolar nerves, thereby improving quality of life and reducing the need for medication [32]. Early results from an ongoing pilot study on percutaneous cryoneurolysis of motor and mixed nerve trunks in spasticity show improvement in the range of motion and patient satisfaction with minimal side effects [28]. These findings exhibit the multifaceted nature of cryoanalgesia in managing chronic pain across multiple conditions. Table 1 outlines the indications and applications of cryoneurolysis in pain management [1,2,5,18-35]. 

**Table 1:** Indications and applications of cryoneurolysis in pain management

Type of pain management	Targeted nerve	Indications	Clinical applications	Study type	Results
Acutepain management	Intercostal nerve	Postoperative incisional pain	Thoracotomy	Case report [18]	Reduced narcotic consumption with simplified nursing care.
		Postoperative surgical pain	Thoracic or thoracoabdominal aortic aneurysm repair	Comparative study [19]	Well-controlled postoperative surgical pain with reduced narcotic usage.
		Acute post-mastectomy pain	Mastectomy	Case report [20]	Reduced pain without opioid analgesics.
		Postoperative pain	Sternotomy	Case report [21]	Reduced Pain with minimal opioid usage.
	Infrapatellar branch of the saphenous nerve	Pain and symptoms associated with knee osteoarthritis	Knee osteoarthritis	Randomized controlled trial [4]	Lower knee pain along with minimal symptoms
		Post-surgical pain	Total knee arthroplasty	Case report [1]	Patients reported pain < 2 on a numeric rating scale of 1-10 with significantly less opioid usage along with lesser time and no gross motor deficits. Skin sensations were returned in four months.
	infrapatellar branch of the saphenous nerve and anterior femoral cutaneous nerve	Post-surgical pain	Total knee arthroplasty	Retrospective study [5]	Significantly lower stay at the hospital, 45% less opioid usage, and reduction in pain intensity.
		Postoperative pain	Total knee arthroplasty	Retrospective study [23]	Improved knee range of motion, ambulation distance, and reduced opioid requirement with no complications.
	Superficial genicular nerves	Postoperative pain	Total knee arthroplasty, severe osteoarthritis	Randomized controlled trial [22]	Reduced opioid consumption and reduced pain
	Suprascapular nerve	Post-surgical pain	Shoulder cuff repair	Case report [1]	Reduced pain with significantly less opioid usage along with less time. Additionally, the full range of motor function was observed two to three weeks post original procedure.
Chronic pain management	Intercoastal nerves	Chronic neuropathic pain	Postherpetic neuralgia	Case report [24]	Achieved sustained pain relief and reduced opioid use.
	Auriculotemporal nerve	Chronic Intractable pain	Temporomandibular joint pain	Retrospective study [25]	Significant improvement in pain scores with a mean number of pain-free months after treatment being seven. Mostly Provided acute pain relief in most and chronic pain relief in some
	Lumbar facet joints	Zygapophyseal joint pain	Lumbar facet joint syndrome	Retrospective study [26]	Reduced pain and depression scores
	Obturator nerve	Pain of obturator neuralgia	Hip adductor spasticity and obturator neuralgia	Case reports [27]	Cryoneurolysis provided excellent long-term pain relief resulting from the spasticity.
	Lateral and/or medial pectoral nerves and the suprascapular nerve.	Spastic pains	Spastic shoulder	Ongoing pilot study [28]	Improved range of motion, spasticity degree, and patient satisfaction at 90 days.
	Anterior femoral cutaneous nerve	Chronic neuropathic pain	Anterior thigh pain	Technical report [29]	Median pain intensity decreased significantly from eight pre-procedure to one post procedure with no complications and lasting up to 12 months follow-up period.
	Greater occipital nerve	Chronic head pain	Occipital neuralgia	Prospective cohort study [30]	Significant pain relief in less than/almost 30 days with an acceptable safety profile.
	Amputated nerve	Chronic phantom limb pain	Amputation	Case report [31]	60% of patients reported 90% to 100% reduction in pain
	Peripheral nerves	Chronic peripheral neuropathy, phantom limb pain	Refractory mononeuropathies, amputation	Quality improvement study [6]	Long-term relief and nerve regeneration.
	Alveolar nerves	Post-surgical chronic dental pain	Dental surgery	Case report [32]	Significant pain relief within three months, reduced usage of pain medications along with improvement in sleep and quality of life.
Oncological pain	Site of neuroma	Chronic recurrent pain post surgical excision	Sural neuroma	Case report [33]	Excellent relief from pain for three months following each serial treatment over a three-year period.
	Quadratus lumborum block	Procedural and post-procedural pain relief	Renal cell carcinoma	Clinical report [34]	Reduced procedural pain
	Tumor infiltrated nerves	Palliative care for pain	Metastatic bone disease and nerve invasion by tumors	Palliative study [35]	Enhanced comfort and quality of life

Oncological pain management

Cryoneurolysis is particularly indicated for neuropathic pain stemming from tumor compression or invasion. Advanced malignancies often compress or infiltrate adjacent nerves, as observed in conditions due to soft tissue or bony tumors or metastasis, such as plexopathies, radiculopathies, and peripheral neuropathies. In these cases, cryoneurolysis provides a localized, opioid-sparing solution by ablating affected nerves, making it a suitable approach for patients who are not viable candidates for surgical resection or where tumor location complicates traditional interventions [38]. This method is usually known as cryoablation. Another key indication is postoperative pain in oncologic surgeries. Procedures that involve nerve manipulation or neural trauma can result in substantial postoperative pain. Cryoneurolysis can offer sustained relief by mitigating nerve-related pain, which complements recovery and reduces reliance on systemic opioids. Additionally, palliative care applications in advanced cancer stages underscore cryoneurolysis’s value in oncologic pain management [35]. For patients with persistent, intractable pain where tumors are non-resectable and cause neuropathic pain, cryoneurolysis serves as an adjunct to systemic analgesics, enhancing the quality of life and providing localized pain control.

The applications of cryoneurolysis in oncologic pain management are diverse, allowing for tailored approaches based on patient needs and clinical contexts. Percutaneous cryoneurolysis is a minimally invasive modality that enables direct access to nerves impacted by tumors, such as in cases of bone metastasis affecting the pelvis or vertebrae. This approach effectively targets neuropathic and somatic pain components without necessitating extensive surgical intervention [35]. Ultrasound-guided cryoneurolysis further refines precision, allowing for the specific targeting of nerves in anatomically complex regions. This technique is advantageous for managing post-surgical pain requiring meticulous nerve localization [33-35].

For palliative care in advanced stages, repeated cryoneurolysis can be applied periodically to sustain pain control in chronic pain syndromes associated with recurrent or progressive disease. This approach is particularly beneficial for reducing or avoiding opioid dependency in patients with pain from metastatic disease affecting spinal or peripheral nerves. In scenarios where visualization is limited, landmark-based cryoneurolysis provides a reliable technique for managing nerve-related pain in hard-to-reach areas, such as the spine or deep pelvic structures, addressing complex metastatic pain that may not respond adequately to conventional treatments [33-35].

Efficacy and safety profile

Hypothesizing the effectiveness of cryoneurological approaches requires examining various pain conditions, including acute and chronic pain, headaches, joint pain, surgical pain, and phantom limb pain. Biel et al. (2022) meta-analyzed these common medical conditions in the general population using cryoneurolysis and interventional radiological techniques. Study data exemplified that cryoneurolysis has a safe profile and reduces opioid consumption [8]. Goyal et al. (2022) examined 10 studies involving 425 patients and found pain alleviation from cryoneurolysis at various follow-up points: seven days, one month, three months, and six months post treatment. The standardized mean difference (SMD) was 1.77 (1.07, 2.46) (P < 0.00001, I² = 79%), 3.26 (2.60, 3.92) (P < 0.00001, I² = 45%), 2.58 (1.46, 3.70) (P < 0.00001, I² = 93%), and 2.38 (0.97, 3.79) (P = 0.001, I² = 86%) respectively at each follow-up point. A higher SMD (on a scale from 0 to 10) indicates a more significant reduction in pain, while an SMD close to 0 suggests little to no effect on pain alleviation. In addition to pain relief, patients also reported improved functional abilities and motor function. No serious complications or side effects were reported, demonstrating the treatment's safety profile [39]. However, a small sample size and substantial heterogeneity in the study findings are limitations, necessitating future trials with a larger sample size and measures to address the heterogeneity [39]. Ye et al. (2021) indicated that cryoneurotherapy can be a part of holistic and integrative medicine in neuropathic pain. Multimodal regimes, including acupuncture, music therapy, radiofrequency ablation (RFA), neuromodulation, and cryoneurolysis, can assist in acute and chronic pain management, owing to their safety and efficacy for many weeks [40].

Wolter et al. (2018) utilized cryoneurolysis for pain management in patients who had multiple cervical facet joint denervations and reported drastic pain relief and no serious side effects other than mild paravertebral muscular discomfort [41]. Lung et al. (2022) concluded that for patients having total knee arthroplasty (TKA), cryoneurolysis is a safe and efficient therapy option that can decrease opioid use, increase knee range of motion, and enhance patient satisfaction [23].

Complications and risk management

Cryo-analgesia risks are similar to those of other needle-based percutaneous procedures, including bleeding, bruising, and infection [42]. Winston et al. (2023) reported that 0.9% (1/113 patients) and 1.78% (2/113 patients) developed local skin infections and bruising or swelling, respectively; all resolved within one month [43]. However, these risks can be eliminated by proper post-procedure care and regular follow-ups.

Specific procedure-related risks include permanent nerve injury, injury to the surrounding tissue, and discoloration of the skin if the cannula is retracted before resolving the ice ball and is allowed to contact other areas near the target site [40]. Although relatedly rare, myonecrosis is a possible complication [44]. To mitigate these risks, cannulas designed specifically for superficial nerves should be used, along with heating units at and below the skin [42].

Cryoneurolysis achieves effective pain management by reversible neuronal injury (Wallerian degeneration). However, once the axon has regenerated, it reconnects with the sensory receptor, and conduction starts again. The regrowth of axons into the perineurium eventually restores sensation, and the block functionally resolves. Hence, there exists little to no chance of a permanent nerve injury, neuroma development, or long-term nerve function changes due to cryo-analgesia [2, 45]. However, Urban et al. (2021) reported that 1.2% (2/169 patients) reported severe dysesthesia in the treatment area, which interfered with their sleep cycle and daily activities, impacting their quality of life [46].

Additionally, studies have reported a rare increase in neuropathic pain following cryoanalgesia during the thoracotomy procedure [47,48]. In these trials, patients reported allodynia, which refers to increased pain perception due to non-noxious stimuli [49]. However, in both trials, analgesia involved surgical exposure and possible nerve retraction. This could potentially be due to afferent barrage during nerve manipulation, leading to hyperalgesia and, subsequently, central sensitization [50]. Preclinical studies indicated that nerve manipulation during cryo-analgesia increases elevated chronic pain risk [51]. Hence, utilizing landmarking techniques and ultrasound instead of traditional fluoroscopy or open surgical methods can help prevent these complications [42].

Comparative analysis

Ilfeld et al. (2020) comprehensively reviewed the role of cryoneurolysis and percutaneous peripheral nerve stimulation in treating acute pain. Cryoablation, RFA, and genicular artery embolization were demonstrated to reduce pain scores dramatically, have good safety records, and can be effectively used as outpatient procedures [45]. Panagopoulos et al. (2023), with the objective of determining the effect of cryoneurolysis and cooled radiofrequency ablation (CRFA) of the genicular nerves for symptomatic pain management in knee osteoarthritis, conducted a randomized controlled trial (RCT) using a diagnostic block of four genicular nerves. Seventy patients with knee osteoarthritis were enrolled and randomized into two groups: a CRFA group with 35 patients and a cryoneurolysis group with 35 patients. The interventions focused on four genicular nerves: the superior medial, superior lateral, inferior medial, and medial (retinacula) genicular branches from the vastus intermedius.

The researchers set out to determine how well CRFA or cryoneurolysis works using the Numerical Rating Pain Scale (NRPS) at 2, 4, 12, and 24 weeks post-intervention. The clinical evaluation utilized the Knee Injury and Osteoarthritis Outcome Score (KOOS), the Oxford Knee Score (OKS), and a seven-point Patient Global Impression of Change (PGIC) scale as secondary outcomes, along with the safety of the two methods. They concluded that cryoneurolysis and CRFA both work to stop the transmission of pain in distinct ways. While cryoneurolysis results in Wallerian degeneration and consequent analgesia, CRFA causes thermal nerve degradation, and the nerve can still recover. Both approaches use identical spherical ablation areas, treatment duration, genicular nerve targeting, and outcome evaluation [52].

Limitations, gaps in evidence, and future directions

Cryoneurolysis has progressed significantly, demonstrating diverse applications in the field of pain management, including oncological pain. However, several limitations and gaps in the evidence hinder the widespread adoption of cryoablation technology. Current studies often have small sample sizes and exhibit significant heterogeneity, which limits the generalizability of the findings. Long-term outcomes and comparative efficacy against other interventional pain management methods, such as RFA, remain underexplored. Furthermore, there is a lack of standardized protocols for cryoneurolysis in different contexts of clinical practice, impeding its application in various acute and chronic pain settings. Although rare, neuropathic pain due to or post cryoneurolysis with surgical manipulation of the nerve remains a significant complication and requires proper protocols for management. 

**Figure 2:**
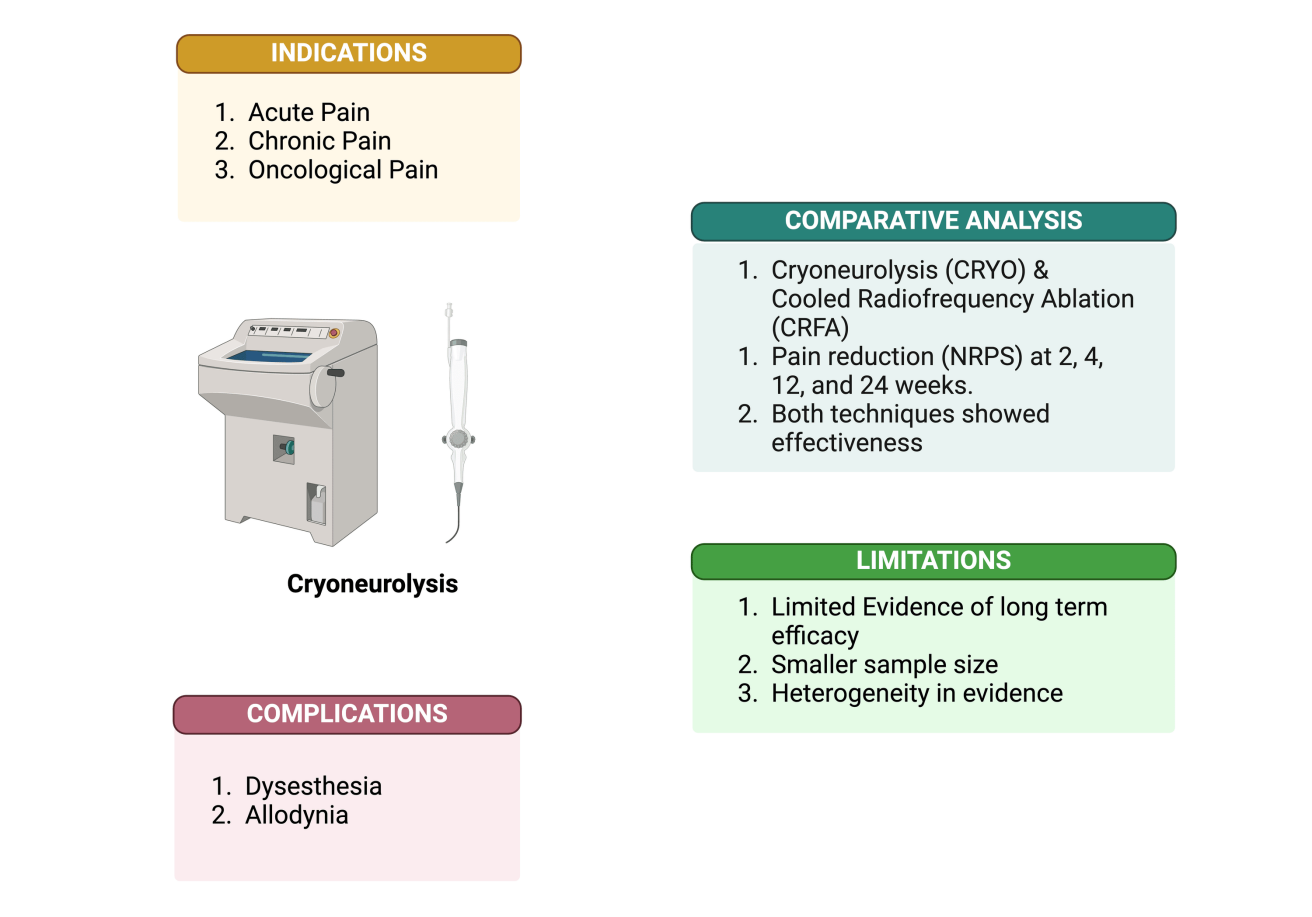
Graphical summary of cryoneurolysis in pain management Created in BioRender. Mylavarapu, M. (2025) https://BioRender.com/k53s484

To address these gaps and limitations, future research should focus on conducting large-scale, multicenter RCTs to establish the safety, efficacy, and long-term outcomes of cryoneurolysis. Furthermore, comparative studies analyzing the efficacy of cryoneurolysis and RFA are needed. Additionally, developing standardized protocols and guidelines for cryoneurolysis across various pain conditions is essential to ensure consistent clinical application. Emerging imaging techniques, such as MRI-guided cryoablation, should also be explored to enhance procedural accuracy and safety, especially in anatomically complex regions. Figure 2 outlines the graphical summary of the review.

## Conclusions

Cryoneurolysis represents a minimally invasive option for managing both acute and chronic pain conditions. This approach maximizes its potential to deliver therapeutic relief while maintaining a lower side effect profile compared to traditional medicinal treatments. The integration of cryoneurology with imaging technology enhances the effectiveness of interventional radiology, making it practical for physicians in both inpatient and outpatient settings. The applicable techniques are reversible, result in minimal collateral tissue damage, offer a favorable safety profile, and strengthen their viability as a treatment strategy for neuropathic pain related to the peripheral nervous system.

The future of cryoneurolysis looks promising, as it suggests a significant role in enhancing pain management techniques. Ongoing research is crucial to fill knowledge gaps and reduce substantial heterogeneity across studies. More comparative trials with larger sample sizes are needed to strengthen statistical evidence, particularly regarding cryoablation and cryoanalgesic methods used in axonal pathways that involve nociceptor desensitization. Technological advancements in imaging guidance, such as the use of MRI, will further improve clarity and broaden the applicability of cryoneurolysis in clinical settings.
